# Direct moxibustion exerts an analgesic effect on cervical spondylotic radiculopathy by increasing autophagy via the Act A/Smads signaling pathway

**DOI:** 10.1002/brb3.2545

**Published:** 2022-03-22

**Authors:** Hui‐Qian Cai, Xin‐Ying Lin, Hai‐Yan Chen, Xi Zhang, Yuan‐Yuan Lin, Shan‐Na Pan, Mei‐Xiang Qin, Sheng‐Yong Su

**Affiliations:** ^1^ Department of Rehabilitation, The First Affiliated Hospital Guangxi University of Traditional Chinese Medicine Nanning Guangxi Province China; ^2^ Department of First School of Clinical Medicine Guangxi University of Traditional Chinese Medicine Nanning Guangxi Province China; ^3^ Department of Nursing, The First Affiliated Hospital Guangxi University of Traditional Chinese Medicine Nanning Guangxi Province China; ^4^ Department of Acupuncture and Moxibustion, The First Affiliated Hospital Guangxi University of Traditional Chinese Medicine Nanning Guangxi Province China

**Keywords:** Act A/Smads signaling pathway, ATG 7 apoptosis, autophagy, cervical spondylosis radiculopathy, direct moxibustion, LC3, nerve regeneration

## Abstract

**Background:**

Direct moxibustion (DM) is reported to be useful for cervical spondylotic radiculopathy (CSR), but the analgesic mechanism remains unknown. Autophagy plays a protective role in neuronal apoptosis, Act A/Smads signaling pathway has been confirmed to be associated with the activation of autophagy. The study aimed to explore the effect of DM on autophagy in rats with CSR and the involvement of Act A/Smads signaling pathway.

**Methods:**

Rats were randomly divided into Sham, CSR, CSR + DM, CSR + DM + 3‐MA (PI3K inhibitor), and CSR + DM + SB (Act A inhibitor) group. Three days after establishment of CSR model with a fish line inserted under the axilla of the nerve roots, DM at Dazhui (GV14) was performed six times once for seven consecutive days. Western blot and immunofluorescence staining were used to observe the expression of the neuronal autophagy molecule LC3II/I, Atg7, and Act A/Smads signaling molecule Act A, p‐Smad2, and p‐Smad3. Bcl‐2/Bax mRNA expression was measured by real time PCR.

**Results:**

DM improved the pain threshold and motor function of CSR rats and promoted the expression of Act A, p‐Smad2, p‐Smad3, LC3II/I, and Atg7 in the entrapped‐nerve root spinal dorsal horn. DM reduced the expression of Bax mRNA and decreased the number of apoptotic neurons. 3‐MA and Act A inhibitor SB suppressed the expression of above‐mentioned proteins and reduced the protective effect of DM on apoptotic neurons.

**Conclusion:**

DM exerts analgesic effects by regulating the autophagy to reduce cell apoptosis and repair nerve injury, and this feature may be related to the Act A/Smads signaling pathway.

## Graphical abstract

Pain threshold and motor function were improved after treatment with Direct Moxibustion (DM) in a rat model of cervical spondylotic radiculopathy (CSR). DM exerts analgesic effects by regulating the autophagy to reduce cell apoptosis and repair nerve injury in rats with CSR. The regulation effect of DM on autophagy may be achieved through activating the Act A/Smads pathway.

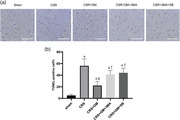

## 1 INTRODUCTION

Neuropathic pain (NP) is the main symptom of cervical spondylotic radiculopathy (CSR) (Expert Panel on Neurological Imaging 2019). NP is characterized by a high prevalence and disability rate and thus seriously lowers the quality of patients and causes a great burden on society (Cohen & Hooten, 2017). Clinical trials (Baron et al., 2010; Jensen & Finnerup, 2014) indicate that the current remedies for NP are not efficacious enough, so further study on the pathophysiological mechanism of NP is necessary, given that its pathogenesis has not been fully clarified. Recent reports (Yang et al., 2020; Yuan & Fei, 2020) showed that autophagy dysfunction can cause nerve cell apoptosis and necrosis, which may lead to neuropathic pain in CSR. Therefore, the regulation of nerve cell autophagy maybe a new target for NP treatment (Romeo‐Guitart & Casas, 2020).

Direct moxibustion (DM) uses its warm and medicinal properties to stimulate people's meridians and collaterals so as to exert a good analgesic effect (Zhou et al., 2017). As an effective therapy, DM has been used in China for more than 4000 years (Li et al., 2021). DM can effectively mitigate neck pain, but its analgesic mechanism has not been fully clarified (Huang et al., 2020; Luo & Fu, 2018; Zhou et al., 2017). Previous study (Cai et al., 2020) found that moxibustion exerts an analgesic effect by regulating neuronal autophagy and reducing cell apoptosis, but the mechanism of how moxibustion regulates autophagy remains unclear.

Activin A (Act A), a member of the transforming growth factor‐beta (TGF‐β) (Xue et al., 2016), is an endogenous neuroprotective factor (Wang et al., 2019). Recent studies (Buchthal et al., 2018; Pettersen et al., 2020; Xue et al., 2017) established that Act A can reduce neuronal apoptosis by increasing the autophagy signal pathway, and the Act A/Smads signal pathway may be involved in the regulation of autophagy in NP. Smads signal activation has also been observed after moxibustion (Kawanami et al., 2020; Zhu et al., 2018). Therefore, we hypothesized that the Act A/Smads signal pathway might be the underlying mechanism regarding how moxibustion regulates autophagy.

This study explores whether DM plays a neuroprotective effect by the trigger Act A/Smads signal pathway and autophagy in a CSR rat model. We also seek to evaluate the relationship between autophagy activation and the Act A/Smads signal pathway.

## 2 MATERIALS AND METHODS

### 2.1 Animals

Eighty adult SPF SD rats (40 females and 40 males, weighing 250 ± 20 g each) were provided by Changsha Tianqin Biotechnology Co., Ltd. (License No.: SCXK [Xiang] 2019‐0014). The rats were fed in the animal room (clean grade) of the First Affiliated Hospital of Guangxi University of Chinese Medicine under natural light, without restriction on drinking water and food. The study protocol was conducted according to the Helsinki Declaration Accords and the guidelines of the Committee on the Care and Use of Laboratory Animals at the Experimental Animal Center of Guangxi University of Chinese Medicine. This study was approved by the Animal Ethical and Welfare Committee of Guangxi University of Chinese Medicine, China (Approval No. DW20190127‐21).

The first 48 rats were randomly divided into three groups: the Sham, CSR, and CSR + DM groups (*n* = 16 per group). CSR group: After the vertebrectomy, the cervical spinal nerve root and dorsal root ganglion were pressed with a fishing line, and 1 ml of 0.9% sodium chloride solution was injected intraperitoneally once a day for 7 days. Sham group: The lamina was opened without placing a fishing line entrapment, and 1 ml of 0.9% sodium chloride solution was injected intraperitoneally once daily for 7 days. CSR + DM group: The rats received direct moxibustion using moxa cones made of *Artemisia argyi* (5 years 5:1 purity produced by Nanyang Hanyi Co., LTD., Nanyang China). A moxa cone (0.5 cm radius and 0.5 cm height of the pillar) was placed directly at the Dazhui (GV14) of CSR rats and then ignited. When 80% of moxa cone had burned, the moxa cone was removed and another cone was placed and ignited. DM was performed six times daily for 7 days.

To examine the potential role of autophagy and Act A signaling pathway activation, the other 32 rats were divided into two groups: CSR + DM + PI3K inhibitors, 3‐methyladenine (3‐MA) and CSR + DM + Act A inhibitor SB‐431542 (SB) (*n* = 16 per group). In the CSR + DM + 3‐MA group, 3‐MA (with a dose of 2.5 mg/kg; MedChemExpress, New Jersey, USA) was injected intraperitoneally 20 min before DM. In CSR + DM + SB group, the Act A inhibitor SB (at a dose of 1 μg/kg; Med Chem Express, New Jersey, USA) was injected intraperitoneally 20 min before DM.

### 2.2 CSR model establishment

All rats were deprived of food and water within 12 h before modeling and weighed and recorded after 12 h. Following the modeling method of Dou et al. (2006), the rats were anesthetized with 10% chloral hydrate at 0.35 ml/100 g. After the rats were fixed in the prone position, the necks were prepared with a shaver, disinfected, and incised for approximately 3 cm from the spinous process of the second cervical vertebra up along the midline of the spine. The subcutaneous tissue and posterior cervical muscle groups were separated layer by layer by blunt dissection to fully expose the left pedicle from C6 to T2 in the field of view, and the surface tissue was scraped. The connective tissue and ligamentum flavum on the C6 to T1 intervertebral space were elevated. After the left pedicle of C7 was gently clamped with nozzle mosquito forceps (pierce‐nosed mosquito forceps), the nylon fishing line (approximately 1.5 cm in length and 0.5 mm in diameter) was gently placed under the nerve roots from C6 to T1 along the longitudinal axis of the spinal cord with microsurgical forceps. After the fishing line placement, the wound was closed layer by layer and sutured. In the Sham group, only the vertebral plate (lamina) was pried open without the placement of the fishing line entrapment.

### 2.3 Behavioral tests

The rats were placed in an observation box between 9:00 and 10:30 every day. After adapting to their surrounding environment for 30 min, the rats were observed as to whether licking, neighing, and other behaviors transpired (Xie et al., 2008). The Kawakami and Dubussion methods (Dubuisson & Dennis, 1977; Kawakami et al., 1994) were used to evaluate gait disorders with foot contracture caused by pain. The rating scale was recorded as follows: 1 = normal gait without forepaw deformity; 2 = normal gait with a marked forepaw deformity such as flexed and/or inverted paw; and 3 = severe gait instability with motor paresis of the ipsilateral left forepaw. The models were made successfully when the score reached two points.

### 2.4 Mechanical hyperalgesia assays

Mechanical hyperalgesia of the front paw was assessed using a YLS‐3E electronic pressure apparatus by referring to the Obata K method (Obata et al., 2004). The blunt plexiglass of the device was vertically stimulated between the third and fourth metatarsi or lateral plantar, and the pressure was gradually increased linearly. Paw withdrawal, shaking, or screaming were considered pain‐like responses, and the pressure value (g) automatically recorded by the instrument was the mechanical pain threshold of the rat.

### 2.5 Western blot

The frozen samples were lysed with RIPA buffer. Their protein concentrations were identified using the bicinchoninic acid assay method. The proteins were subjected to sodium dodecyl sulfate polyacrylamide gel electrophoresis on a 10% gel and transferred onto polyvinylidene fluoride membranes. Membranes were blocked with 5% (w/v) bovine serum albumin in Tris‐buffered saline with Tween 20 for 1 h. These membranes were incubated using P‐Smad2 antibody (1:1000, Affinity), anti‐P‐Smad3 antibody (1:800, Affinity), anti‐Atg7 antibody (1:1200, Affinity), anti‐LC3II /I antibody (1:800, Affinity), anti‐ACTA antibody (1:800, Santa Cruz), and anti‐β‐actin (1:1000, Affinity) and were diluted in Tris‐buffered saline with Tween 20 buffer overnight at 4°C. The immunoblots were then incubated with peroxidase‐conjugated secondary antibody (1:3000; ZB2305, ZSGB‐BIO, Beijing, China) for 90 min at room temperature. Band intensities obtained by western blot assay were determined using the ImageJ open‐source software (National Institutes of Health, Bethesda, MD, USA).

### 2.6 Real‐time qRT‐PCR assay

Total RNA was extracted using the Trizol method and reverse transcribed into cDNA with a reverse transcription kit. Using cDNA as a template, the PCR reaction system was configured with a SYBR Green fluorescent quantitative PCR kit. Real‐time PCR amplification was performed on a fluorescent quantitative PCR instrument. Reaction program: pretreatment at 95°C for 30 s with PCR cycles (40 cycles at 95°C for 5 s, 60°C for 30 s, 72°C for 15 s). The Beclin‐1/Bcl‐2 primers and product length were 1, and the primers were synthesized by Shanghai Sangon Biological Engineering Co., Ltd. The relative expression of the target mRNA was analyzed by the 2‐Ct method using β‐actin as an internal reference.

### 2.7 Immunofluorescence

Tissue sections were deparaffinized and high‐pressure repaired with citric acid. Fresh 3% H_2_O_2_ was prepared with distilled water or PBS, and the tissues were blocked at room temperature for 5–10 min and washed three times with distilled water. Normal goat serum blocking solution was added dropwise for 20 min at room temperature. Excess liquid was shaken off. The dripped diluted two primary antibody working solutions were used overnight at 4°C, and the sections were removed the next day after returning to room temperature and washing three times with PBS for 5 min each time. The corresponding mixed working solution of fluorescent secondary antibody was added dropwise and was washed three times in PBS after 20 min at 37°C in the dark. The DAPI staining solution was incubated at room temperature in the dark for 20 min. Then, a water‐soluble mounting medium was mounted. The intracellular distribution and content of Act A and LC3 were observed by fluorescence microscopy. To complete immunofluorescence data analysis, the Integrated Density of LC3 and Act A were quantified in all samples by Image J.

### 2.8 Transmission electron microscopy

Spinal cord tissues were fixed in 1% glutaraldehyde for 2 h, hydrated in a graded alcohols and amyl acetate, and embedded in SPI‐Pon812. Ultrathin (60−80 nm) sections were produced on an ultramicrotome and then stained using uranyl acetate and lead citrate. Finally, ultrathin sections were examined using a HT7700 electron microscope (Hitachi, Tokyo, Japan).

### 2.9 TUNEL assay

Tissues from entrapped nerve roots and spinal cord were harvested after 7 days of intervention and cut into thin slices for the TUNEL assay. The assay was performed using the TUNEL assay kit according to the manufacturing instructions (Roche). Observations were made using a fluorescence microscope (Olympus) for the TUNEL percentage of the number of positive cells.

### 2.10 Statistical methods

SPSS 21.0 software was employed to analyze and process the experimental data. Normally distributed measurement data were expressed as mean ± standard deviation, as multiple sample means were compared using analysis of variance and subsequent pairwise comparisons of means were performed using the LSD method. *p*<.05 was considered the criterion for a statistically significant difference.

## 3 RESULTS

### 3.1 Direct moxibustion increases the pain threshold in the CSR rats

**FIGURE 1:**
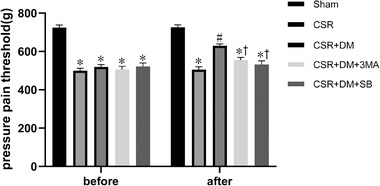
Pain threshold rats before and after intervention. *p<.05, vs. sham group; #*p* <.05, vs. CRS group. DM, direct moxibustion; CSR, cervical spondylotic radiculopathy

The results are presented in Figure 1. Relative to the Sham group, the pain thresholds of the model and DM groups were significantly reduced before the intervention (*p* < .05). After the intervention, the pain threshold in the DM group relative to that of the model group increased distinctly (*p *< .05).

### 3.2 Direct moxibustion improves the motor function of CSR rats

**FIGURE 2:**
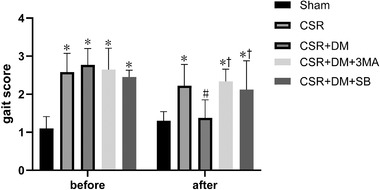
Motor function of rats before and after intervention. *p<.05, vs. sham group; #*p* <.05, vs. CRS group; +*p* <.05, vs. CSR + DM group. DM, direct moxibustion; CSR, cervical spondylotic radiculopathy

As shown in Figure 2, the gait scores for the model and DM groups were markedly higher prior to intervention (*p* < .05) than those of the sham group. Relative to the model group, the DM group showed a significant decrease in gait scores after intervention (*p* <.05).

### 3.3 Direct moxibustion enhances the expression of autophagy in spinal dorsal horn of CSR rats

**FIGURE 3:**
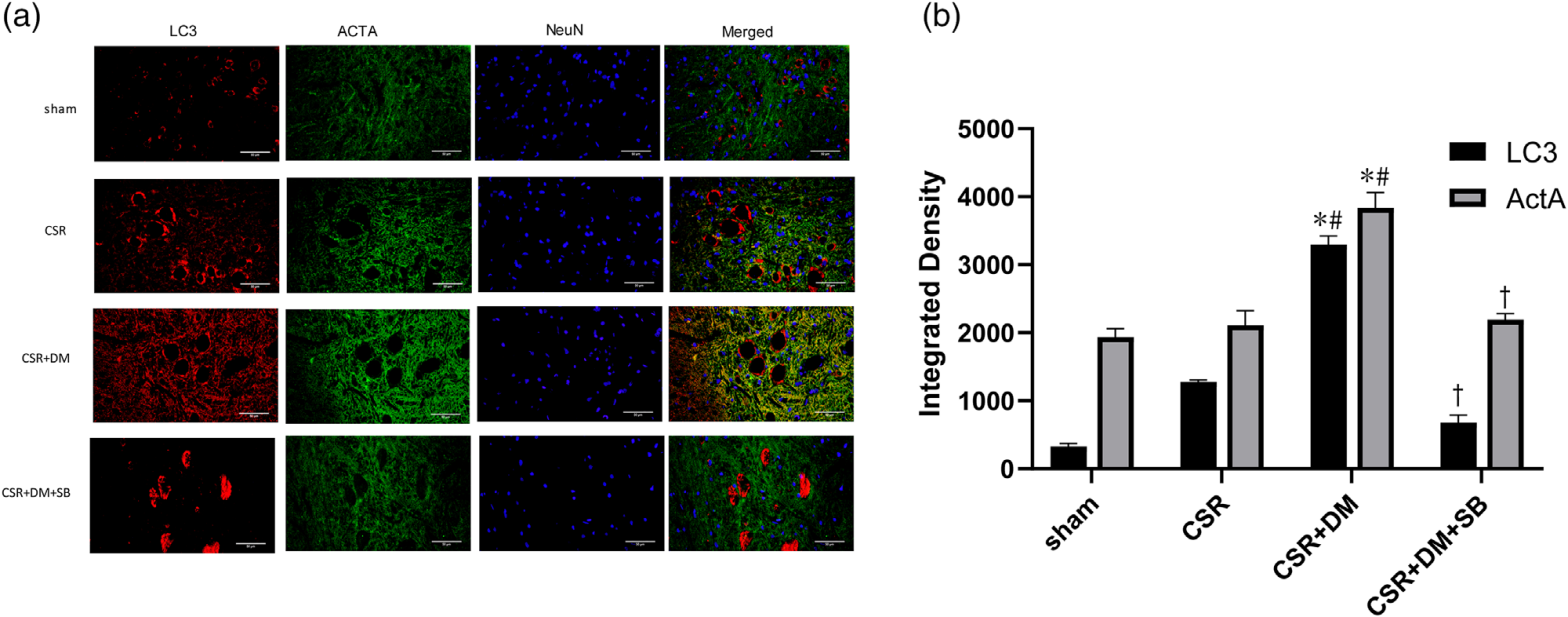
Immunofluorescence staining of LC3 and Act A expression in the spinal dorsal horn at 7 days after intervention. (a) Representative LC3/Act A photomicrographs from the spinal dorsal horn at 7 days after intervention. (b) Integrated density quantification of LC3 and ACT A expression. The densities of the protein bands were analyzed and normalized to β‐actin fluorescence colors; LC3: red, Act A: green, and nucleus:blue. Scale bars: 50 μm. *p < .05, vs. sham group; #*p* < .05, vs. CRS group; +*p* < .05, vs. CSR + DM group. SB, Act A inhibitor SB‐431542; DM, direct moxibustion; CSR, cervical spondylotic radiculopathy

**FIGURE 4:**
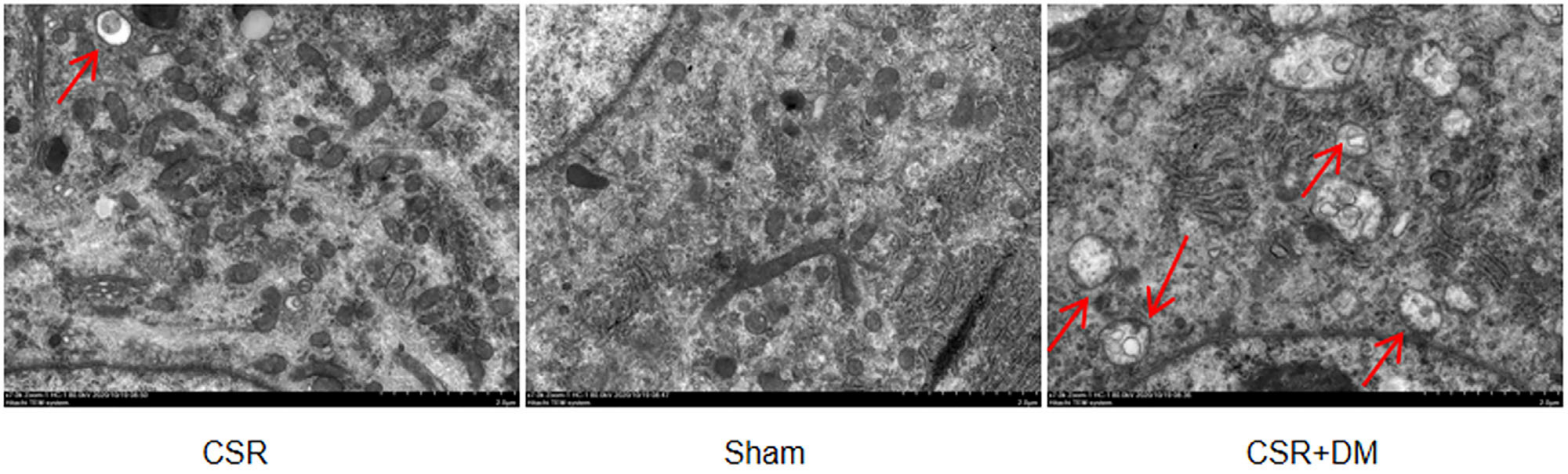
Ultrastructure of neurons in the spinal dorsal horn at 7 days after intervention. DM, direct moxibustion; CSR, cervical spondylotic radiculopathy. Red arrow: autophagosome. Scale bars: 2 µm

LC3 is a reliable marker for the detection of autophagy induction in mammalian cells. Immunofluorescence analysis revealed that the staining density of LC3 in the CSR group was lower, and that of LC3 was significantly higher in the CSR + DM group (Figure 3). More autophagic vesicles also formed in the CSR + DM than in the CSR group as detected under the transmission electron microscope (Figure 4).

### 3.4 Direct moxibustion activates autophagy and the Act A/Smads signaling pathways in spinal dorsal horn neurons of CSR rats

**FIGURE 5:**
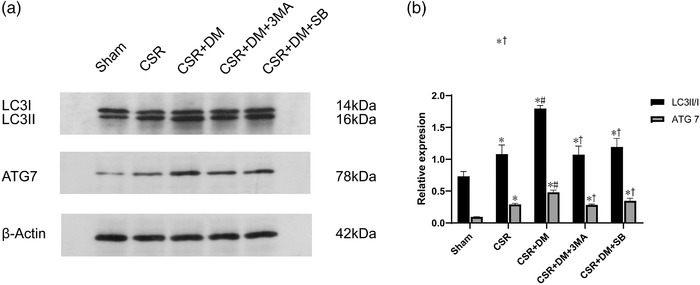
LC3II/LC3I, ATG7 protein expression in the spinal dorsal horn at 7 days after intervention. (a) Western blot assay of LC3II/LC3I, ATG7 protein. (b) Relative protein band densitier of LC3II/LC3I, ATG7. The densities of the protein bands were analyzed and normalized to β‐actin. * *p*<.05, vs. sham group; #*p* <.05, vs. CRS group; +*p* <.05, vs. CSR + DM group. SB, Act A inhibitor SB‐431542; 3‐MA, PI3K inhibitor; DM, direct moxibustion; CSR, cervical spondylotic radiculopathy

**FIGURE 6:**
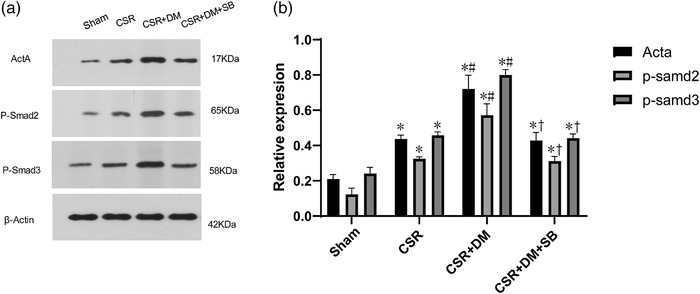
Act A, P‐Smad2, P‐Smad3 protein expression in the spinal dorsal horn at 7 days after intervention. (a) Western blot assay of Act A, P‐Smad2, P‐Smad3 protein. (b) Relative protein band densities of Act A, P‐Smad2, P‐Smad3. The densities of the protein bands were analyzed and normalized to β‐actin. *p<.05, vs. sham group; #*p* <.05, vs. CRS group; +*p* <.05, vs. CSR + DM group. SB, Act A inhibitor SB‐431542; DM, direct moxibustion; CSR, cervical spondylotic radiculopathy

Immunofluorescence analysis showed that the staining density of Act A was weak in the sham group. However, the density was higher in the CSR and the CSR + DM groups (Figure 3). Western blot analysis (Figures 5 and 6) revealed that relative to the counterparts with the sham and CSR groups, the levels of Act A, p‐Smad2, and p‐Smad3 proteins in the Act A/Smads signaling pathway and autophagy‐related LC3II/I and Atg7 proteins in the CSR + DM group were significantly increased after the DM treatment (*p* < .05). This outcome suggested that DM therapy activated the expression of autophagy and the Act A/Smads signaling pathways after CSR.

### 3.5 Effect of direct moxibustion on the activation of autophagy is attenuated by Act A inhibitor SB

To explore the effect of Act A/Smads signaling pathway on the autophagy of neurons, CSR rats were injected with Act A inhibitor SB in advance. After joining SB, the levels of autophagy related factors LC3II/I and Atg7 protein in the CSR + DM + SB group were significantly decreased compared with those in the CSR + DM group (*p* < .05; Figure 5).

### 3.6 Direct moxibustion inhibits the apoptosis of spinal cord nerve cells in CSR rats

**FIGURE 7:**
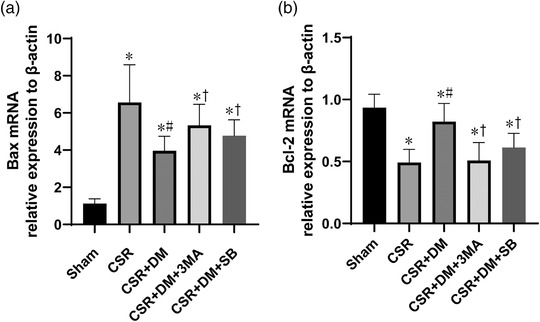
Bcl‐2 mRNA, bax mRNA expression in the spinal dorsal horn at 7 days after intervention. *p<.05, vs. sham group; #*p* <.05, vs. CRS group; +*p* <.05, vs. CSR + DM group. SB, Act A inhibitor SB‐431542; DM, direct moxibustion; CSR, cervical spondylotic radiculopathy

**FIGURE 8:**
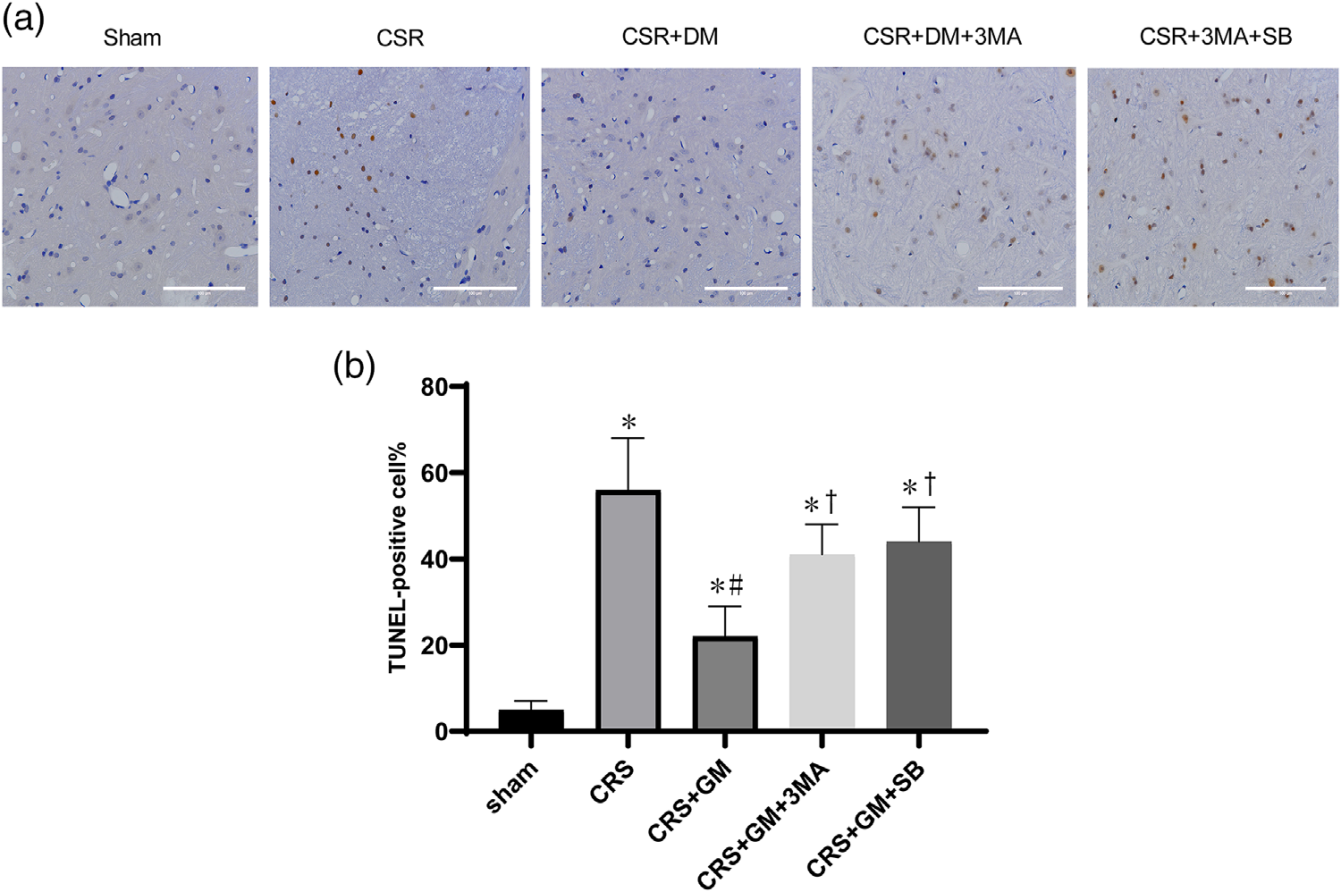
Neuron apoptosis in the spinal dorsal horn at 7 days after intervention. (a) TUNEL assay was used to detect neuronal apoptosis. (b) Quantitative results of ratio of TUNEL‐positive cells and the number of degenerating neurons. *p<.05, vs. sham group; #*p* <.05, vs. CRS group; +*p* <.05, vs. CSR + DM group. SB, Act A inhibitor SB‐431542; 3‐MA, PI3K inhibitor; DM, direct moxibustion; CSR, cervical spondylotic radiculopathy

To investigate the effect of direct moxibustion on the apoptosis of spinal cord nerve cells in CSR rats, the mRNA expression levels of Bcl‐2 and Bax in spinal homogenate were evaluated by real‐time PCR after 7 days of intervention. The Bcl‐2mRNA levels of the CSR group were significantly lower than those of the sham group (*p* < .05; Figure 7). However, the DM treatment improved the inhibition of Bcl‐2mRNA expression caused by CSR modeling (*p* < .05; Figure 7). At the same time point, the level of Bax mRNA in the CSR group was significantly higher than that in the sham group but was decreased by DM treatment (*p* < .05; Figure 7). TUNEL analysis further confirmed this conclusion. After the operation, the cell apoptosis in the spinal cord of rats increased (*p* < .05; Figure 8). The intervention of DM improved the apoptosis in the rats with CSR and reduced the apoptosis rate by 34% compared with the model group (*p* < .05; Figure 8). All of these outcomes suggested that DM treatment could reduce the apoptosis of nerve cells after CSR by preventing the decrease of the antiapoptotic pathway and blocking the activation of the proapoptotic pathway.

### 3.7 Effect of direct moxibustion on the inhibition of nerve cell apoptosis is blocked by 3‐MA and Act A inhibitor SB

To investigate the role of autophagy activation and Act A/Smads signal transduction in neuronal apoptosis after the intervention of DM, the DM‐treated rats were injected with 3‐MA or Act A inhibitor SB. As a PI3K inhibitor, 3‐MA can block the later stage of autophagy and inhibit the formation of autophagosomes. In the CSR + DM + 3‐MA group, preadministration of 3‐MA inhibited the enhanced effect of DM on LC3 and ATG7 in the CSR + DM group (*p* <.05; Figure 5). Meanwhile, the antiapoptotic effect of DM was offset by 3‐MA. Compared with the outcome in the CSR + DM + 3‐MA group, the antiapoptotic protein Bcl‐2 was decreased and the proapoptotic protein Bax was increased in the CSR + DM + 3‐MA group (*p* < .05; Figure 7). The comparable effects were also observed in the CSR + DM + SB group. Meanwhile, the antiapoptotic effect of DM was offset by 3‐MA. Compared with the outcome of the CSR + DM group, the Bcl‐2mRNA was decreased, but the Bax mRNA and apoptosis rate were increased in the CSR + DM + 3‐MA group (*p* < .05; Figure 7). Comparable effects were also observed in the CSR + DM + SB group. The experimental results proved that both 3‐MA and Act A inhibitor SB could weaken the antiapoptotic effect of DM on nerve cells.

## 4 DISCUSSION

Following the theory of traditional Chinese medicine (TCM), moxibustion is a type of external treatment like acupuncture and usually bakes acupoints with burning moxa wools (Deng & Shen, 2013). Burning moxa produces thermal and radiation effects, converts physical signals to biological counterparts, and exerts an analgesic effect by activating endogenous reparation and the protection of the human body (Chen et al., 2020; Wu et al., 2016). Moxibustion has been widely applied in pain‐related diseases, including cervical spondylosis (Li, 2020), lumbar disc herniation (Chen, 2021), and central NP (Zhang et al., 2017). NP is one of the major domains of moxibustion analgesia research (Lee et al., 2010; Shao, 2014).

The study of NP pathogenesis has been a hot spot for a decade (Bannister et al., 2020). Many studies prove a close relationship between the occurrence of NP and cell autophagy (Jin et al., 2018; Piao et al., 2018) and that nerve cell autophagy dysfunction is an important cause of NP. The activation of autophagy leads to critical material degradation in cells to release ATP, thereby alleviating the cytoplasmic metabolic process of an energy crisis and avoiding cell apoptosis and necrosis (Corti et al., 2020; Mizushima & Levine, 2020). Therefore, modest activating autophagy nerve cell expression is the key to the treatment of CSR NP.

Studies have shown that moxibustion regulates autophagy expression. Li et al. (2021) found that moxibustion could protect the heart function of rats with heart failure by regulating autophagy through the mTOR signaling pathway. Another research (Zhang et al., 2019) revealed that moxibustion improved the ratio of LC3‐ II/LC3‐I so as to regulate the function of autophagy in the cerebral cortex and hippocampus of mice and enhanced the removal of abnormal deposits in the cerebral cortex and hippocampus Aβ1‐42 in mice. Previous results (Cai et al., 2020) established that autophagy activation after moxibustion treatment played a noticeable role in neuronal protection and motor recovery in the rats of CSR.

In this study, we focused on the effect of direct moxibustion intervention on nerve cells and the mechanism of its regulation of autophagy. We found that a DM intervention enhanced the formation of autophagosomes in nerve cells, and the ratio of LC3‐II/LC3‐I and the expression of Atg7 protein were increased. Nucleation and expansion of the autophagosome require LC3‐ I lipidation to LC3‐II mediated by various conjugation reactions by ATG proteins. A cytosolic form of LC3 (LC3‐I) is activated by ATG7, and then conjugated to phosphatidylethanolamine to form LC3‐II, which is recruited to autophagosomal membranes. Therefore, the ratio between LC3‐II and LC3‐I is considered a biomarker of autophagosome formation (Galluzzi & Green, 2019; Martin‐Rincon et al., 2018). Wang et al. (2018) also discovered that moxibustion increased the ratio of LC3II/LC3I in the substantia nigra of rats with Parkinson's disease and enhanced the autophagic clearance of nerve cells.

In addition, TUNEL detected that the apoptosis of spinal nerve cells in CSR rats was significantly less than that in the model group after the intervention of DM. To further clarify the antiapoptosis effect of DM, we detected the contents of Bax and Bcl‐2 in the spinal cord tissue. Bax and Bcl‐2 are common apoptosis‐regulating proteins in the Bcl family (Peña‐Blanco et al., 2018). During cells apoptosis, Bax adheres to the mitochondrial membrane to induce apoptosis (Campbell & Tait, 2018; Stevens et al., 2019).

In contrast to Bax, Bcl‐2 inhibits cell apoptosis by forming a dimer with Bax. DM inhibits the expression of proapoptotic signal Bax induced by nerve compression and increased the expression of antiapoptotic factor Bcl‐2. Yang et al. (2021) found that moxibustion increased the expression of Bcl‐2 protein and decreased the expression of Bax protein in the brain of aging rats.

To further explore the relationship between autophagy and apoptosis, we added autophagy inhibitor 3‐MA before the treatment of DM and confirmed that the antiapoptosis effect of DM was significantly inhibited. Thus, DM might reduce nerve tissue damage by blocking the apoptotic signaling pathway, and its antiapoptotic effect might be attributed to the activation of autophagy, an outcome that is consistent with the study of Zhang et al. (2019) Therefore, activation of autophagy can be used as a strategy for the NP reduction, and DM could be a potential treatment.

As an endogenous neuroprotective factor, Act A plays an important protective role in neurodegenerative and neuropsychiatric diseases through the Act A/Smads signaling pathway (Buchthal et al., 2018; Su et al., 2018). External injury factors can induce the high expression of Act A in nerve cells and activate the phosphorylation and activation of Samd 2/3, thereby regulating the expression of target genes, prolonging the survival of neurons, and alleviating neuronal injury (Magga et al., 2019). Recent studies (Xue et al., 2016; Zhang et al., 2017) verified that exogenous Act A reduced the apoptosis of nerve cells by increasing autophagy during nerve damage. It is speculated that the Act A/Smads signaling pathway may be involved in the regulation of autophagy in NP. The results of our study showed that after DM intervention, the expressions of Act A, p‐Smad2, and p‐Smad3 in the Act A/Smads pathway could be increased, but after inhibiting the expression of Act A, the expressions of autophagy factors LC3 and Atg7 were also inhibited, as consistent with prior research (Pettersen et al., 2020). The DM activation of autophagy may be regulated by the Act A/Smads signaling pathway.

Our study still has some limitations. First, the experiment was done in vivo and the data were relatively limited. Second, we only confirmed that the activation of autophagy by DM was related to the Act A/Smads signaling pathway, but the direct mechanism is still unclear. Despite these limitations, this work is a preliminary study on treating CSR rats with DM and provides a theoretical basis for studying the analgesic mechanism of DM in the treatment of CSR.

## 5 CONCLUSIONS

In conclusion, pain threshold and motor function were improved after the intervention of DM in a CSR rat model. The exploration of this mechanism showed that DM has a positive effect on nerve cell repair and activates the Act A/Smads signal pathway and autophagy to promote neuronal repair and inhibit cell apoptosis. Therefore, the activation of neuronal autophagy is related to the Act A/Smads signaling pathway.

## FUNDING INFORMATION

This study was supported by the National Natural Science Foundation of China (Grant no. 81960895) and the Guangxi Natural Science Foundation (Grant no. 2018GXNSFAA281111).

## AUTHOR CONTRIBUTIONS

SYS conceived and designed the study. HQC and XYL performed the experiments, collected, and analyzed the data, and wrote the manuscript. HYC, XZ, YYL, SNP, and MXQ collected and analyzed the data. All the authors agreed for submission and approved the final version of the manuscript accepted for publication.

## CONFLICT OF INTEREST

The authors declare no conflicts of interest.

### PEER REVIEW

The peer review history for this article is available at https://publons.com/publon/10.1002/brb3.2545.

## Data Availability

The data that support the findings of this study are available from the corresponding author upon reasonable request.
